# A GLO10 score for the prediction of prognosis in high grade gliomas

**DOI:** 10.18632/oncotarget.20195

**Published:** 2017-08-10

**Authors:** Feng Chen, Peng Peng, Yi Zhou, Zhen-Yu Yang, Hai-Quan Zhang, Xiang-Sheng Ao, Da-Quan Zhou, Chun-Xiang Xiang

**Affiliations:** ^1^ Department of Neurosurgery, Xiangyang Central Hospital, Hubei University of Arts and Science, Xiangyang, Hubei 441021, P. R. China; ^2^ Department of Pathology, Xiangyang Central Hospital, Hubei University of Arts and Science, Xiangyang, Hubei 441021, P. R. China

**Keywords:** glioblastoma, prognosis, biomarker, DNA microarray, LASSO

## Abstract

Gliomas are the most common lethal brain tumours and remain great heterogeneity in terms of histopathology and clinical outcomes. Among them, glioblastomas are the most aggressive tumours that lead to a median of less than one-year survival in patients. Despite the little improvement of in diagnosis and treatments for last decades, there is an urgent need for prognostic markers to distinguish high- and low-risk patients before treatment.Here, we generated a list of genes associated with glioblastoma progressions and then performed a comprehensive statistical modelling strategy to derive a 10-gene (GLO10) score from genome wide expression profiles of a large glioblastoma cohort (n=844). Our study demonstrated that the GLO10 score could successfully distinguish high- and low-risk patients with glioblastomas regardless their traditional pathological factors. Validated in four independent cohorts, the utility of GLO10 score could provide clinicians a robust prognostic prediction tool to assess risk levels upfront treatments.

## INTRODUCTION

Glioma is among the most common brain tumour type. With median survivals of less than a year for patients, glioblastoma is considered as a vital cause of cancer mortality in both adults and children despite aggressive surgery, chemotherapy and radiation [1, 2]. By the year of 2007, according to the World Health Organization (WHO), gliomas were assessed based on histopathological features and categorized into astrocytomas, oligodendrogliomas, mixed oligoastrocytomas, and ependymomas [3]. However, limitations of this grading system were addressed, such as high rate of inter- and intra-observer variability [4, 5]. Therefore, in 2016, molecular signature was introduced in diagnosing tumours due to the explosion of genomic information during the last decade [6].

DNA microarrays were introduced two decades ago. As this technology provides more comprehensive and objective information than traditional microscopic morphology, it has revolutionized cancer research. To date, there are molecular based diagnostic tests using DNA microarrays. Some have been incorporated in clinical practice guidelines, including Mamma Print and Oncotye Dx [7]. A number of DNA microarray based studies have identified prognostically distinct molecular subtypes of gliomas [8–12]. These approaches were based on unsupervised hierarchical or k-means clustering of genes. As reported in these studies, patients belonging to various subgroups (clusters) showed significantly different prognostic outcomes independent to some known clinical factors, such as age and grade. However, the clustering-based prognostic signatures remain challengeable in clinical practice as the results were dramatically affected by the selected genes. Previous studies remain inconsistency in the prognostic gene candidates, which increases the difficulties in making such methods practical applicable.

Here, we generated a 10-gene (GLO10) score for the prediction of glioblastoma overall survival from DNA microarray expression datasets. We trained the prognostic models in a large cohort (n=470) and demonstrated that the GLO10 score successfully distinguished high- and low-risk groups in other three independent cohorts comprising patients with glioblastomas (n=374). Our results unveiled novel glioma prognostic biomarkers that could be easily applied with great potential in producing robust results in clinical practices.

## RESULTS

### Identification of glioblastoma associated genes

**Figure 1:**
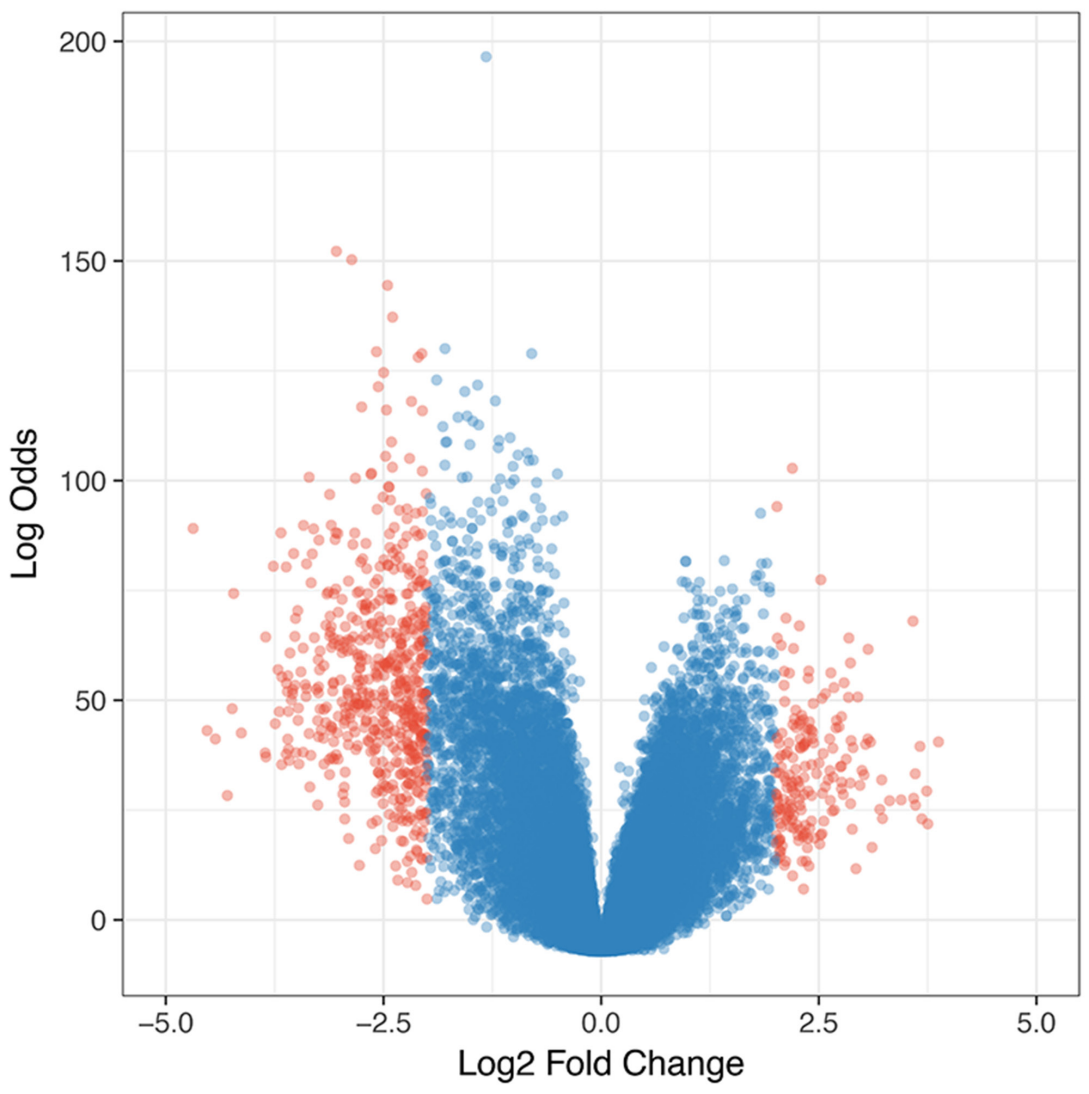
Volcano plot of differentially expressed genes in glioblastomas The red dots indicate those were considered as significant up- and down- regulated genes.

**Figure 2:**
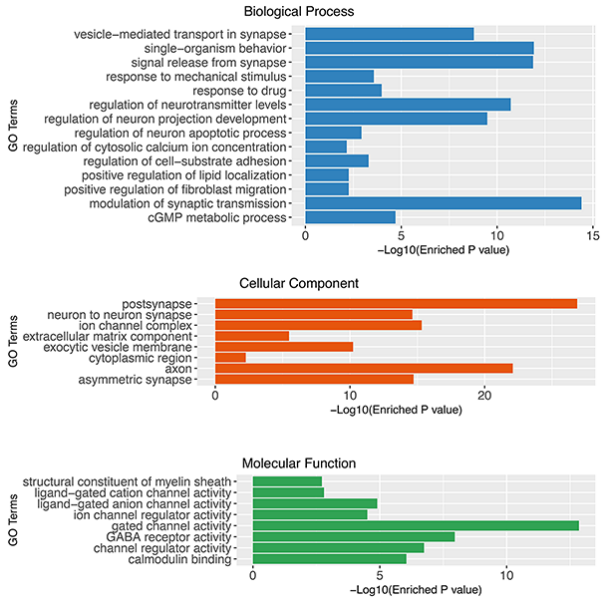
Gene set enrichment analysis of glioblastoma associated genes using Gene Ontology (Cellular Component, Biological Process and Molecular Function)

**Figure 3:**
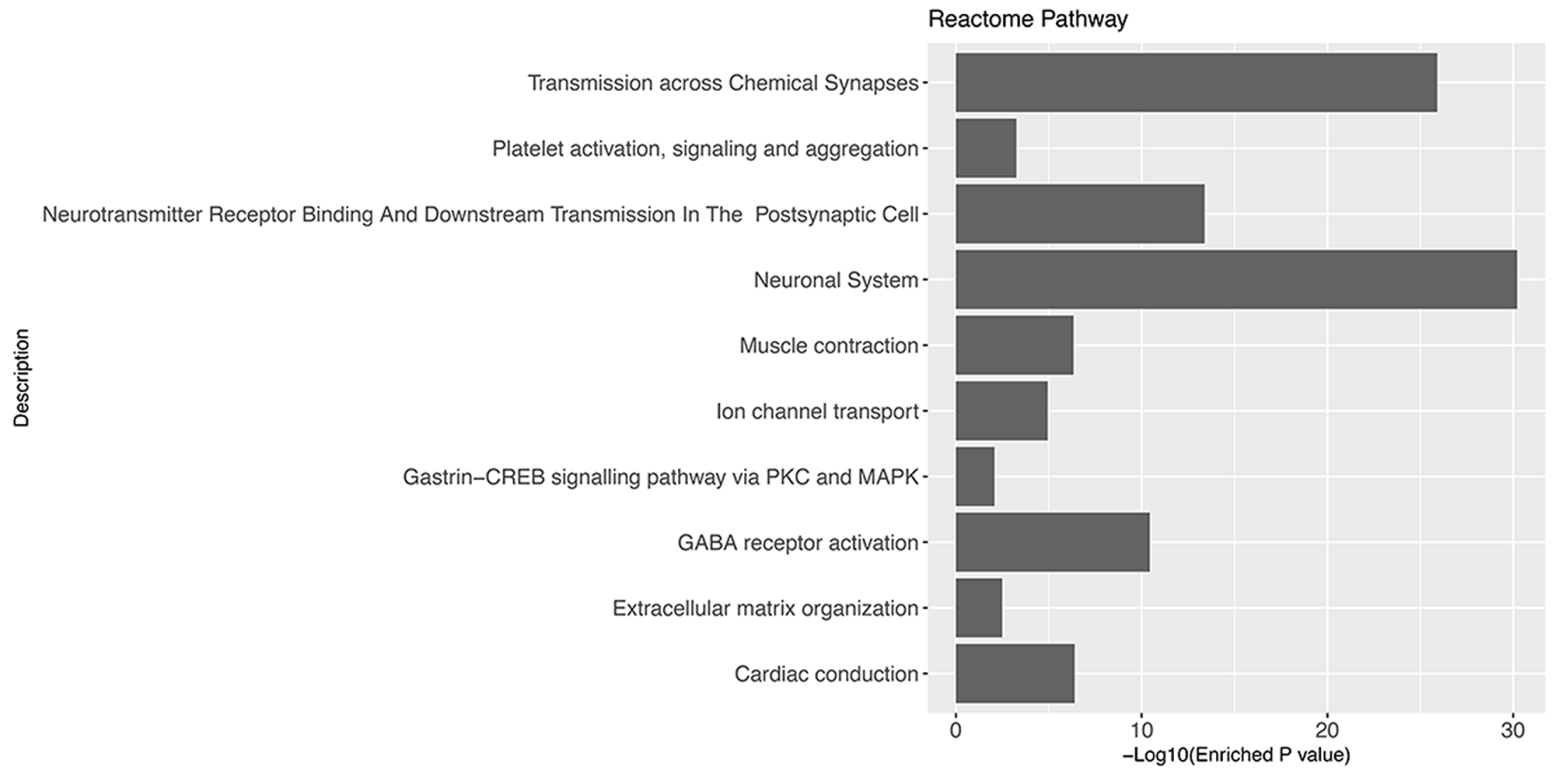
Gene set enrichment analysis of glioblastoma associated genes using Reactome Pathway database

We hypothesized that the prognostic biomarkers were associated with tumour genesis and progression. In this case, the glioblastoma associated genes were identified by comparing gene expression profiles of tumour tissues and normal brain samples. The data was retrieved from one previously published large scale study (n= 256), Repository of Molecular Brain Neoplasia Data (Rembrandt)[13]. After data preprocessing and differential expression analyses, a total of 723 probe sets corresponding to 552 genes were identified as significantly differentially expressed genes (DEGs) in glioblastomas. Among the DEGs, 137 were up-regulated and 415 were down-regulated (Figure 1). The gene functional analyses revealed that the glioblastoma associated genes were significantly enriched in the Gene Ontology Cellular Components including postsynapse (P=1.36×10^-27^), axon(P=8.14×10^-23^), ion channel complex(P=4.75×10^-16^), asymmetric synapse(P=1.90×10^-15^), neuron to neuron synapse(P=2.33×10^-15^), exocytic vesicle membrane(P=5.92×10^-11^), extracellular matrix component (P=3.34×10^-06^) and cytoplasmic region (P=5.43×10^-03^); Biological Processes including modulation of synaptic transmission(P=3.94×10^-15^), regulation of neurotransmitter levels(P=1.94×10^-11^), regulation of neuron projection development(P=3.24×10^-10^), regulation of cell-substrate adhesion(P=5.05×10^-04^), positive regulation of fibroblast migration(P=5.49×10^-03^); Molecular Functions including gated channel activity(P=1.143×10^-13^), GABA receptor activity(P=8.95×10^-09^), channel regulator activity(P=1.45×10^-07^), calmodulin binding(P=7.21×10^-07^), ligand-gated anion channel activity(P=1.02×10^-05^), ion channel regulator activity(P=2.50×10^-05^), ligand-gated cation channel activity(P=1.27×10^-03^) and structural constituent of myelin sheath(P=1.54×10^-03^)(Figure 2). The pathway enrichment analysis (Figure 3) revealed that the identified glioblastoma associated genes were enriched in various pathways that involved in glioblastoma progression, for example GABA receptor activation [14], Ion channel transport [15, 16] and Extracellular matrix organization [17–19].

### Univariate prognostic analysis by Cox proportional hazards models

To investigate the prognostic potentiality, we integrated a larger multi-institutional data set of 470 glioblastoma patients from the TCGA cohort (Discovery set). Previously identified glioblastoma associated genes were fitted into Cox promotional hazards models. Based on the overall survival association significance assessed by log rank test, these probe sets were ranked and filtered at the cutoff of P < 0.001. A total of 28 probe sets were selected for statistical modelling.

### LASSSO statistical modelling to derive GLO10 score

**Figure 4:**
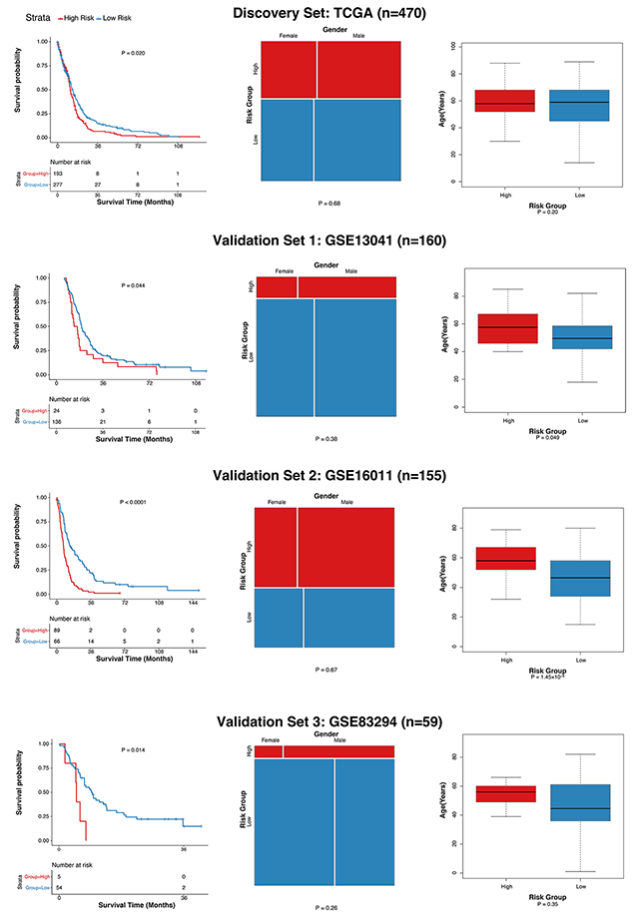
Evaluation of the association of GLO10 score with glioblastoma survival Left: Kaplan-Mert plot of high- and low-risk groups, P value was calculated using the log-rank test. Middle: Comparison of gender ratios in high- and low-groups, P value was calculated using Pearson’s chi-squared test. Right: Comparison of the distributions of age in high- and low-risk groups, P value was calculated using Wilcoxon rank-sum test.

A statistical regression algorithm based on the least absolute shrinkage and selection operator (LASSO) was applied to relate the 28 probe sets to the patient survival in the discovery set. We have optimized a 10-gene (Table 2) signature (GLO10) as the weighted sum of expression levels of the 10 genes for each patient. High GLO10 scores were significantly associated with shorter overall survival times. After the parameter optimization, we have categorized the patients into high-, or low-risk groups based on the GLO10 score threshold of 3.58. The high- and low-risk groups showed significant differences in terms of overall survival (Figure 4, Table 1).

**Table 1:** Clinical characteristics of discovery and validation sets

Discovery Set: TCGA				
Characteristics	**Overall**	**High risk group**	**Low risk group**	P-value
	(n=470)	(n=193)	(n=277)	
Median overall survival[months](range)	10.37 (0.10-127.55)	10.03 (0.10-127.55)	10.58 (0.10-108.81)	0.020^§^
Female [n (%)]	181 (38.51%)	77 (39.90%)	104 (37.54%)	0.68^‡^
Median age[years](range)	59.00 (5.00-89.00)	58.00 (10.00-88.00)	59 (5.00-89.00)	0.20^†^
**Validation Set 1: GSE13041**				
Characteristics	**Overall**	**High risk group**	**Low risk group**	P-value
	(n=160)	(n=24)	(n=136)	
Median overall survival[months](range)	12.84 (0.23-110.22)	9.04 (1.74-72.02)	13.58 (0.23-110.22)	0.044^§^
Female [n (%)]	63 (39.38%)	7 (29.17%)	56 (41.18%)	0.38^‡^
Median age[years](range)	51.50 (18.00-86.00)	57.50 (40.00-85.00)	49.50 (18.00-86.00)	0.049^†^
**Validation Set 2: GSE16011**				
Characteristics	**Overall**	**High risk group**	**Low risk group**	P-value
	(n=155)	(n=89)	(n=66)	
Median overall survival[months](range)	8.76 (0.24-150.72)	7.08 (0.24-66.72)	14.16 (0.48-150.72)	<0.0001^§^
Female [n (%)]	50 (32.26%)	27 (30.34%)	23 (34.85%)	0.67^‡^
Median age[years](range)	55.00 (14.00-80.00)	58.00 (14.00-79.00)	46.50 (15.00-80.00)	1.45×10^-6†^
**Validation Set 3: GSE83294**				
Characteristics	**Overall**	**High risk group**	**Low risk group**	P-value
	(n=59)	(n=5)	(n=54)	
Median overall survival[months](range)	7.80 (0.23-41.00 )	5.03 (1.74-7.80)	9.29 (0.23-41.00)	0.014^§^
Female [n (%)]	32 (54.24% )	1 (20.00%)	31 (57.41% )	0.26^‡^
Median age[years](range)	47.00 (1.00-82.00 )	56.00 (39.00-66.00)	44.50 (1.00-82.00)	0.35^†^

‡ P value calculated using the Pearson’s chi-squared test.

† P value calculated using the Wilcoxon rank-sum test.

§ P value calculated using the log-rank test.

**Table 2:** List of gens in the GLO10 score

Probeset ID^§^	Symbol	Gene ID*	Chromosomal location	Description
221898_at	PDPN	10630	1p36.21	podoplanin
202133_at	WWTR1	25937	3q25.1	WW domain containing transcription regulator 1
203706_s_at	FZD7	8324	2q33.1	frizzled class receptor 7
201792_at	AEBP1	165	7p13	AE binding protein 1
221766_s_at	FAM46A	55603	6q14.1	family with sequence similarity 46 member A
202718_at	IGFBP2	3485	2q35	insulin like growth factor binding protein 2
203729_at	EMP3	2014	19q13.33	epithelial membrane protein 3
212063_at	CD44	960	11p13	CD44 molecule (Indian blood group)
203504_s_at	ABCA1	19	9q31.1	ATP binding cassette subfamily A member 1
201761_at	MTHFD2	10797	2p13.1	methylenetetrahydrofolate dehydrogenase (NADP+ dependent) 2, methenyltetrahydrofolate cyclohydrolase

§ Affymetrix Human Genome Array probe set identifier.

* NCBI Gene Database https://www.ncbi.nlm.nih.gov/gene.

To assess the robustness of the GLO10 score, we then evaluated its performance in other three independent glioblastoma cohorts with GEO accessions GSE13041 (validation set 1, n=160), GSE16011 (validation set 2, n=155) and GSE83294 (validation set 3, n=59). Patients with GLO10 scores higher than 3.58 were assigned into the high risk group, while those with GLO10 scores lower than the threshold were considered as low risk. As shown in Figure 4, there are significant differences between high-risk and low-risk groups (validation set 1: P = 0.044, validation set 2: P < 0.0001, validation set 3: P=0.014). Surprisingly, although high-risk patients tended to have higher age, the gender ratio showed insignificant different patterns (Figure 4, Table 1 ). This indicated that the GLO10 score retained prognostic power independent of other traditional factors, such as age and gender. The dominance of GLO10 score in the prognosis prediction revealed the importance of the 10 genes (Table 2) in glioblastoma diagnosis and treatment researches.

## DISCUSSION

For the prognostic biomarker discovery in glioblastomas, there remains discordance among scientific publications given various sample requirements, data complexity, evolving technologies and lack of golden standard practice guidelines [7, 20]. However, due to high complexity of genomics in glioma patients, considerable challenges still present in implementing such strategies. To address these challenges, we applied statistical modelling approaches and leveraged large scale of gene expression profiles from multi-institutional cohorts. The robustness of the GLO10 score was demonstrated by successfully implemented in more than three independent cohorts.

Functional analyses revealed that genes consisting the GLO10 score were involved in the genesis and progression of glioblastomas (Figures 2 and 3). Among them, the prognostic associations with glioblastoma outcomes have already been reported. For example, Insulin-like growth factor-binding protein 2 (IGFBP2) has been considered as a glioma oncogene [21]. Increasing expression of IGFBP2 could associated to poor glioma prognosis as it may play major role in glioma tumour progression [22–27]. It is also reported that in IDH-mutant glioma, IGFBP2 was inhibited so that patients’ survival could be improved [28]. The expressions of FZD7, along with other two genes, SFRP1 and SFRP4, were identified to be associated with poor prognosis in glioma patients [29]. The up-regulated expression of FZD7 could promote glioma cell proliferation [30]. However, to our understanding, GLO10 the first gene signature that utilize the expression data and sum into a unified score that ease its applications in clinical practices.

For the future work, we will collect more datasets and re-train the model to improve the robustness and predictive power. We would also assess its prognostic value by comparing other biomarkers such as IDH1 and 1p19q status. In summary, our analysis of data from different independent cohorts demonstrates that the utility of GLO10 score as a tool for glioblastoma prognosis prediction. The incorporation of the GLO10 into the prognosis prediction for newly diagnosed glioma patients will facilitate the development of biomarker and drug target discovery.

## MATERIALS AND METHODS

### Datasets collection and preprocess

In this study, we collected datasets from various cohorts including The Cancer Genome Atlas (TCGA)[31], Repository for Molecular Brain Neoplasia Data (Rembrandt)[13] and other published studies. Gene expression profiling datasets and clinical information of five independent cohorts were retrieved NCBI GEO Database with accession numbers: GSE68848 (Rembrandt cohort), GSE83130 (TCGA cohort), GSE13041 [11], GSE16011 [32] and GSE83294 [8]. Given the complexity of data sets, we restricted the samples to grade three and four glioblastomas in this study.

For the Rembrandt cohort, a total to 28 normal and 228 glioblastoma samples were considered. For the glioblastoma prognostic gene signature training, the numbers of samples considered for this study were 470, 160, 155 and 59 for the TCGA cohort (discovery set), GSE13041 (validation set 1), GSE16011 (validation set 2) and GSE83294 (validation set 3). While searching for the data sets, Affymetrix HG-U133A and HG-U133 Plus 2.0 microarrays were considered. Since they are sharing 22,277 probe sets, the performances of the derived gene signatures could be easily evaluated in different studies without gene ID mapping.

The raw fluorescence intensity profiles (*.CEL) were preprocessed, background corrected and normalized with RMA algorithm [33] using Bioconductor package, *affy* [34], in the R environment.

### Differential gene expression analysis

We applied the differentially expressed gene analysis by using the *limma* (linear models for microarray data, [35]) algorithm. All the gene expression data was converted into the log base-2 scale before comparison using the Welch's *t*-test with Benjamini & Hochberg correction [36]. The functional analysis was based on the gene set enrichment analysis in Gene Ontology and Reactome Pathway database [37].

### Statistical modelling

For the development of prognostic gene signature, we used the datasets from the discovery set (TCGA cohort, n=470). A linear regression modelling based on the LASSO algorithm was implemented by the *glmnet* [38] R package. Briefly, the *glmnet* package fits a generalized linear model via penalized maximum likelihood and extract the prognostic gene signature by10-fold cross validation approaches. A subset of 10 genes was selected as their weighted combined gene expression data was significantly correlated to the overall survival outcomes of glioblastoma patients in the discovery set.
